# A Consideration of Some Problems in a Dentist’s Life

**Published:** 1895-08

**Authors:** Ezra F. Taft

**Affiliations:** Cambridge, Mass.


### A CONSIDERATION OF SOME PROBLEMS IN A DENTIST'S LIFE.[1]

BY EZRA F. TAFT, D.M.D., CAMBRIDGE, MASS.

A STUDENT once put the question to me, "Do you, after many years of practice, feel that you are still learning by experience something new to help you in receiving patients: reading their whims, disposition, and character, and trying to make such an impression upon them as shall harmonize the interests of both?"

Like the parson who preached a sermon on the devil, and gave an exhaustive view of his text because he was full of it, so I am prepared to speak exhaustively, if not exhaustingly, upon a subject in which I am so much interested. I once knew of a professional man who aimed to make his life an easy one. In his daily routine of dental practice his time was fully occupied when in his office, though his hours were so limited that at the close of the day he did not feel he had been physically or mentally pressed beyond his limit.

Ill health caused a dentist occupying apartments with him to retire from business and offer his practice for sale. The temptation was too great. My friend saw here an opening which promised good results, and undertook what was really the work of two men, hardly realizing what new cares and responsibilities he was assuming. Then with his old friends still seeking him, and the crowding in of patients from his new field, he soon found it was one thing to have a large practice and another to be able so to regulate it as to feel that he was not physically or mentally pressed beyond the proper limit. The case of this man working harder than he ought is common to the profession generally. In other words, our practice dominates our lives rather than that we regulate and control our practice in such a way that our health does not suffer, and that we leave ourselves time for recreation. I shall consider this question first from the stand-point of the patient, and then from that of

---

[1] Read before the Harvard Odontological Society, September 27, 1894.

the practitioner. It is impossible to classify our patients except in a general way. The varieties are as infinite as the characteristics of the human family.

We stand at the door of some practitioner and interview the different ones as they enter the reception-room. Our purpose is to make a study of them, learning, if possible, in what mood they have come there, and asking point-blank why they have been led to make this call upon their dentist.

We see at once that education has had its influence. It makes no difference whether it is a practitioner in an exclusive locality in one of our large cities, or in a wooden business block of a back country town. If he is a dentist taking such a stand as will do honor to his profession, his callers will be the rich and the poor, the educated and the ignorant, those who appreciate good work, and those who are so distrustful as to ask many questions before even telling why they have called. They are callers, but not all patients. If a man's time is fully occupied he will naturally work for those patients who most appreciate his work, and are willing to pay what it is worth. Fortunate is he if he can employ some assistant to relieve him of multitudinous cares, and especially to see all visitors. The average practitioner has no such assistant, perhaps not even a servant to answer the bell and show the patient in.

An early appointment to accommodate some one who is going away finds your first patient a little late on account of early household duties. You begin your work for her, however, when some one steps in just for a moment to speak of one of the many hundred things that seems little to the patient, but which is a certain amount of hinderance to the practitioner.

Next, a gentleman calls on the way to his business to make an appointment for his wife. A lady has entered; the rustling of paper in her hand gives you encouragement that a bill is to be paid, and you gladly step in to see her. Your surmise is correct, but before handing you the money she begs you to look at your books to make sure there was not a mistake in the number of fillings indicated in the bill. She is sorry to detain you, but it will take only a minute, and she will be better satisfied.

A nervous, fidgety woman steps in next, to say, with a voice heard almost in the street, that she had not worn that under set of teeth hardly an hour since they were put in. They would not stay in place, and her mouth was too sore for anything.

A little girl runs in to tell you that her mother was obliged to go away and couldn't keep her appointment the next day, and, after taking several minutes in studying the appointment-book, another time is set. A self-important young man, in joyous costume of latest cut, holding a lighted cigarette between his fingers, would like to know if you had any leisure time this morning, as his wisdom-tooth was giving him a great deal of trouble.

If you are in full practice it is a great help to you, and is money well expended, to have some kind of assistant; yet even then it is often necessary for the dentist himself to see the caller. Many reserve certain parts of the day for consultation, examination, etc.; yet I do not believe it is possible to carry out that plan, as but few patients would learn of that rule, and when known it would be almost impossible to confine themselves to certain hours. You are in a profession depending upon the public for your practice, and to the public you must cater. If your time is fully occupied, you would be glad to put up a sign on your door as to whom you would like for patrons; but that also is impossible. Your callers are from all classes. You must be, at least, civil to every one. Fortunate is that man whose practice is not a local one, but whose patients seek him from miles around, and who makes many of his appointments through the mail. We speak of a man who, through good work and a good name, has secured a large practice, and has the most of his regular office hours filled with appointments for at least two or three weeks in advance.

The dentist who is in full practice can have no respite during the working hours of the day, and it is not simply mechanical labor, but a continuous effort of the mental and physical powers of the operator. He will work for one patient only a short time, whose nervous organization is such as to try the most patient man. He will work for another a full half day and, on account of the equanimity and serenity of this person, will be but very little tired, and fully equal to the work required of him the rest of the day. Standing, as many do, from eight or nine in the morning till five in the afternoon, with one or at most two hours' intermission at noon, and not only this, but meeting and working for all classes and conditions of men: children, whose eyes and actions show an uncontrollable fear; women, whose nervousness stands out in their very voice; men, whose time is so valuable that their impatience is almost unendurable. Under these varied circumstances what is to be expected? There may be a constitution now and then made of iron; there may be a nervous organization so wiry that no amount of twisting and pulling can harm it; there may be one occasionally who has the happy faculty of so directing his work that it leaves no bad results; but the average man cannot do this. With his time all occupied he finds himself at the close of the day a mental and physical sufferer. Let this be kept up and the overstrained nerves will finally yield. Physically, there are few professions calling for such constant strain on one's system. The enforced and unnatural position which must be sustained for so long a time will introduce us to the pangs of rheumatism, lumbago, and kindred ills. The strain on the eyes, which is so constant, brings on a series of head troubles, from which very few of us are free. All this means that the average practitioner is doing more than he is able, and does not know any method of lightening his labors and still hold the practice he desires to have. It means that he is not master of his own time, but a slave to his patients and friends. You, gentlemen, know that I am stating no exaggerated case, however it seems to those out of the profession.

Three brothers were comparing notes on their different occupations,—the one a bank-teller, the second a physician, the third a dentist,—each claiming his life was a struggle, and more undesirable than the others.

The teller, especially, laid stress on the fact that his time was not his own; that his hours were defined, his work was to be done every day, with no cessation. Whatever his physical feelings, or his longing for a little outing, he was under the control of a higher power that said "No."

The physician felt he was a burdened man. To be sure he was his own master; but his life-work was to battle with disease, and as he had the care of the health of different families, he had great responsibility; he must be ready to go night or day, and have but very little leisure or time that he could calculate on as being his own.

The dentist's life was looked upon by the other two as most attractive by comparison: perfect control of his time by appointment, making his day as long as he saw fit, drawing such a line among patients as to exclude those who were undesirable, and, finally, so remunerative,—large fees with but very little expense.

Doubtless this is the way our profession is regarded by many who look only upon the surface, but the busy dentist could quickly convince them of their mistake, and prove to them that his calling ranks among the most wearing of occupations, physically and mentally, made so in part by petty annoyances to which I have before alluded. If, then, the daily routine of our professional life is such that not only is our time taken up with regular appointments, but we are usually overcrowded by reason of the many interruptions which must come in every man's practice, we must conclude that we are bowing ourselves too much to the will of our patients; we are not free men to do with our time as we would wish to. We have our intimate friends, our old patients,—yes, new ones whom we are anxious to hold, who appear before us and ask our favors. The solution of the problem is a difficult one. You may say the remedy lies in two ways,—have some one to assist you, or be wilfully independent. I cannot believe the assistant will very much relieve the situation, and such independence is contrary to our nature. Let us, then, accept the situation, that while we are professional men we are also Yankees,—after the almighty dollar. We seek for honor, and wish to be held in esteem by the public and our professional brethren; but we also wish to make a living, do something more than simply pay our expenses. Very natural, then, when overcrowded with patients, to try and accommodate all, to work early and late rather than let some slip from our grasp. Under these circumstances what must the dentist be to withstand all this and meet with success? He must be a lineal descendant of both Job and Sampson. He must be firm, and yet sympathetic; be able to so control the mind of his patients that they will not worry him; be amiable, and woe to him who loses his temper. Good humor will sell more goods, build the best houses, plead the most successful cases, write the best sermons, and perform the best dental operations. Some one has said, "A melancholy musician may compose a dead march, but he cannot storm the castle of the soul with the rhythmic artillery of lovely light and joy." A melancholy poet may write Dante's "Inferno," but cannot give us Milton's "Paradise Lost." The world is full of music. The man who can sing and will not sing ought to be sent to Sing Sing.

> "It is easy enough to be pleasant
> When life flows by like a song;
> But the man worth while is the one who will smile
> When everything goes dead wrong."

If it is impossible to curtail our work while in the office, let us do all we can to recuperate when out of it. Let us ride some hobby. In change we find the most perfect rest. Don't forget the couplet of Oliver W. Holmes,—

> "Run if you like, but try to keep your breath.
> Work like a man, but don't be worked to death."

